# Intelligence-Aware Batch Processing for TMA with Bearings-Only Measurements

**DOI:** 10.3390/s21217211

**Published:** 2021-10-29

**Authors:** Gabriele Oliva, Alfonso Farina, Roberto Setola

**Affiliations:** 1 Department of Engineering, Università Campus Bio-Medico di Roma, 00128 Rome, Italy; r.setola@unicampus.it; 2 Selex-ES (retired), 00144 Rome, Italy; alfonso.farina@outlook.it

**Keywords:** target motion analysis, intelligence-aware estimation, radar, Cramér–Rao lower bound, nonlinear estimation, constrained MLE, data fusion, smart estimation, intelligence analysis, critical infrastructure protection, evolutionary ant colony optimization, MIDACO-SOLVER

## Abstract

This paper develops a framework to track the trajectory of a target in 2D by considering a moving ownship able to measure bearing measurements. Notably, the framework allows one to incorporate additional information (e.g., obtained via intelligence) such as knowledge on the fact the target’s trajectory is contained in the intersection of some sets or the fact it lies outside the union of other sets. The approach is formally characterized by providing a constrained maximum likelihood estimation (MLE) formulation and by extending the definition of the Cramér–Rao lower bound (CRLB) matrix to the case of MLE problems with inequality constraints, relying on the concept of generalized Jacobian matrix. Moreover, based on the additional information, the ownship motion is chosen by mimicking the Artificial Potential Fields technique that is typically used by mobile robots to aim at a goal (in this case, the region where the target is assumed to be) while avoiding obstacles (i.e., the region that is assumed not to intersect the target’s trajectory). In order to show the effectiveness of the proposed approach, the paper is complemented by a simulation campaign where the MLE computations are carried out via an evolutionary ant colony optimization software, namely, mixed-integer distributed ant colony optimization solver (MIDACO-SOLVER). As a result, the proposed framework exhibits remarkably better performance, and in particular, we observe that the solution is less likely to remain stuck in unsatisfactory local minima during the MLE computation.

## 1. Introduction

In the last decades, target motion analysis (TMA) has become an increasingly popular research field, and in the literature, several approaches have been developed, such as batch processing frameworks [1,2,3,4] and recursive ones [3,5,6,7,8]. The aim of TMA is to estimate the state of a target (usually position and velocity) from noise-corrupted measurements collected by an observer [9]. The TMA problem presents several challenges, mainly due to the nonlinear relationship between the measurements and target state. Another challenge is that the observer must outmaneuver the target in order to make the target state observable [10]. For instance, to track a target with constant velocity, the observer platform must change its speed or course. Otherwise, there exist other target trajectories that produce the same sequence of noise-free bearing angles [3].

Among other approaches, bearing-only target tracking [11,12,13] represents an increasingly popular topic, with application scenarios ranging from underwater tracking [14,15] to cooperative tracking for multiagent systems [15,16,17,18].

Other relevant approaches in the literature include, among other works: applications to sensor network localization [19]; algorithms based on direction-of-arrival measurements, modeled by von Mises–Fisher distributions [20]; pseudolinear estimators for 3D target motion analysis by a single moving ownship collecting azimuth and elevation angle measurements [21]; a methodology based on a bank of batch maximum a posteriori (MAP) estimators as a general estimation framework that provides the relinearization of the entire state trajectory, multihypothesis tracking and an efficient hypothesis generation scheme [22]; an approach based on Newton–Raphson methods and Particle Swarm Optimization [23]; a methodology to combine target motion compensation and track-before-detect methods within passive radar based on global navigation satellite systems (GNSS) for the detection of maritime targets [24]; TMA from cosines of conical angles acquired by a towed array [25]; and a new pseudolinear filter for bearings-only tracking without the requirement of bias compensation [26].

Notice that, in the literature, some approaches have been developed where the availability of road or traffic information is used to track a moving target. In particular, in [27], the target is assumed to move in a a road network, and the tracking is performed via an airborne sensor that exploits knowledge on the network; in [28], a similar setting is considered, and a Bayesian approach is adopted; in [29], a particle filter is developed in order to track multiple vehicles on multi-lane roads based on a microscopic traffic flow model. However, such approaches require one to rely on a large deal of fine-grained information (e.g., the structure of the road network) and can only be applied to scenarios involving roads. However, in many cases, especially considering a maritime context, only coarse-grained information is available: for instance, the environment might contain physical obstacles or deterring entities such as warships that discourage the target from passing nearby, or there might be rough evidence of the presence of a target in a given zone (e.g., due to a witness or to cheap range-free sensors able to only detect the presence of a target in a given zone). In this view, relying on such a coarse-grained information could help improve the target’s trajectory estimate, also in contexts where road network information cannot be leveraged upon without requiring huge computational resources. This has been demonstrated, for instance, in [30] where such information is used in the framework of network localization to overcome localization ambiguities.

This is the aim of this paper. In particular, this paper considers a scenario where additional information is available to the ownship in charge of estimating the target’s trajectory; specifically, the ownship is aware that the trajectory of the target lies in the intersection of some sets and is not contained in the union of some other sets. This additional information is exploited by developing a constrained MLE problem and an approach for the selection of the ownship’s trajectory mimicking the Artificial Potential Fields technique [31,32], which is typically used by mobile robots to aim at a goal (in this case, the region where the target is assumed to be) while avoiding obstacles (i.e., the region that is assumed not to intersect the target’s trajectory). Moreover, from a theoretical standpoint, the CRLB on the estimation covariance matrix is characterized in the case of MLE problems with inequality constraints; this is performed by extending the approach in [33,34], where equality constraints where discussed, via the cast of inequality constraints into nonsmooth equality ones and by the adoption of generalized Jacobian matrices [35], which are set-valued on a zero-measure set where the derivative of the resulting nonsmooth function is not defined.

The paper is complemented by an experimental analysis showing the effectiveness of the proposed approach.

To summarize, the main contributions of the paper are as follows:

We develop a novel MLE approach to carry out batch target-tracking estimation based on noisy bearing-only measurements, which incorporates as inequality constraints additional information in terms of sets where the target’s trajectory is assumed to be contained and other sets which have empty intersection with the target’s trajectory;
We characterize the CRLB associated to the constrained problem by considering a generalized set-valued Jacobian matrix of the constraints function and by resorting to nonsmooth theory;
We provide a heuristic way to select the ownship’s trajectory based on the although coarse-grained available information regarding the target’s trajectory.

## 2. Materials and Methods

The aim of this section is to present the main algorithms, tools and derivations that are used in the paper. Specifically, the section is organized as follows: in Section 2.1, we present our problem statement; then, we discuss maximum likelihood estimation problems (in Section 2.2, we review the unconstrained case, while in Section 2.3 we develop the proposed MLE approach with additional inequality constraints); Section 2.4 is devoted to addressing the computational aspects related to the approximated solution of the above constrained and unconstrained MLE problems; Section 2.5 and Section 2.6 address, respectively, the characterization of the CRLB in the unconstrained and constrained case; finally, Section 2.7 discusses a heuristic approach to choosing the ownship’s direction based on the available information.

### 2.1. Problem Statement

Let us consider a scenario where a target moves in a linear motion on a plane; in particular, considering a discrete-time sampling, let us assume that the target moves according to the following equations:

(1) $$\begin{array}{cc} x_{t}\left(k\right) & =x_{t0}+{\dot{x}}_{t0}kT+\frac{1}{2}{\ddot{x}}_{t}k^{2}T^{2}\\ y_{t}\left(k\right) & =y_{t0}+{\dot{y}}_{t0}kT+\frac{1}{2}{\ddot{y}}_{t}k^{2}T^{2}\\ {\dot{x}}_{t}\left(k\right) & ={\dot{x}}_{t0}+{\ddot{x}}_{t}kT\\ {\dot{y}}_{t}\left(k\right) & ={\dot{y}}_{t0}+{\ddot{y}}_{t}kT, \end{array}$$

with *T* being the sampling time. Moreover, let us consider an ownship platform aiming to estimate the parameter vector

(2) $$\psi ={\left[\begin{array}{cccccc} x_{t0} & y_{t0} & {\dot{x}}_{t0} & {\dot{y}}_{t0} & {\ddot{x}}_{t} & {\ddot{y}}_{t} \end{array}\right]}^{T},$$

based on a batch of measurements, sampled at uniform discrete-time instants t=kT during the ownship motion and evaluated over the time interval $\left[0,k_{\max}T\right]$.

In more detail, we assume that the ownship attempts to sense the following *nominal measurement* (e.g., see [36]):

(3) $$h\left(\psi ,k\right)=\mathrm{atan}2\left(y_{t}\left(k\right)-y_{o}\left(k\right),x_{t}\left(k\right)-x_{o}\left(k\right)\right)$$

where $x_{o}\left(k\right),y_{o}\left(k\right)$ are the coordinates of the ownship at time t=kT along the x and y axes, respectively. However, we consider a scenario where the ownship is actually provided with noisy measurements with the following structure:

z(k)=h(ψ,k)+w(k),

where the terms $w\left(k\right)∼N\left(0,\sigma^{2}\right)$ are independent and identically distributed Gaussian noises with zero-mean and variance $\sigma^{2}$.

Further to that, let us assume that additional information is available; specifically, let us assume that the ownship is aware that, during the considered time frame $\left[0,k_{\max}T\right]$, the position $p\left(k\right)={\left[x_{t}\left(k\right),y_{t}\left(k\right)\right]}^{T}$ of the target is confined in a region P of the plane defined as follows:

(4) $$P=\left\{p\in R^{2}\;|\;p\in \overset{m}{\underset{i=1}{⋂}}X_{i}\right\},$$

where the sets $X_{i}\subseteq R^{2}$ are convex, and their intersection is nonempty; moreover, the ownship is aware that the position p(k) lies outside a region S of the plane defined as follows:

(5) $$S=\left\{p\in R^{2}\;|\;p\in \overset{r}{\underset{i=1}{⋃}}Y_{i}\right\},$$

where the sets $Y_{i}\subseteq R^{2}$ are convex.

The aim of this paper is to investigate how this additional information influences the estimation of θ and how the ownship can leverage on this additional information to select a trajectory that improves the estimation performance.

### 2.2. Maximum Likelihood Estimation without Additional Information

Let us discuss how to estimate the parameter vector ψ via the maximum likelihood estimate (MLE) technique (see, for instance [37], p. 182). The MLE for a vector parameter ψ is defined to be the value $\theta^{*}$ that maximizes the likelihood function $p(z_{1},z_{2},\ldots ,z_{m},\theta )$ over the allowable domain of θ. In what follows, where understood, we abbreviate the notation by writing p(θ). When p(θ) is differentiable, we have that the MLE $\theta^{*}$ satisfies

(6) $$\frac{\partial \ln(p(\theta ))}{\partial \theta }{|}_{\theta =\theta^{*}}=0_{n}.$$

It is worthwhile to mention that the solution of the above equation is unique in this case and is theoretically asymptotically unbiased. For our case, we have the following expression for the likelihood function [3]:

(7) $$\begin{array}{cc} p(\theta ) & =\frac{1}{2\pi \sigma }\prod_{k=1}^{k_{\max}}\exp\left(-\frac{{\left(z\left(k\right)-h\left(\theta ,k\right)\right)}^{2}}{2\sigma^{2}}\right). \end{array}$$

Notably, the maximization problem at hand can be formulated as

(8) $$\theta^{*}=\arg\max_{\theta }p\left(\theta \right)=\arg\max_{\theta }\ln\left(p\left(\theta \right)\right).$$

Let us define λ(θ)=−ln(p(θ)). The above problem can be equivalently expressed as

(9) $$\theta^{*}=\arg\min_{\theta }\lambda \left(\theta \right)$$

which, by simple computations is equivalent to solving [3]

(10) $$\begin{array}{cc} \theta^{*} & =\arg\min_{\theta }\sum_{k=1}^{k_{\max}}\frac{{\left(z\left(k\right)-h\left(\theta ,k\right)\right)}^{2}}{2\sigma^{2}}. \end{array}$$

Notably, Equation (10) is recognized to be the classical least squares (LS) solution.

### 2.3. Maximum Likelihood Estimation with Additional Information

Let us now extend the above MLE framework in order to account for the additional intelligence available to the ownship.

In particular, we notice that, by Equations (1) and (2), it holds that

(11) p(k)=Q(k)ψ,

with

(12) $$Q\left(k\right)=\left[\begin{array}{ccc} I_{2} & kTI_{2} & \frac{1}{2}k^{2}T^{2}I_{2} \end{array}\right]\in R^{2\times 6}.$$

Therefore, by plugging the above expression in Equation (4), we have that p(k)∈P if and only if

(13) $$Q\left(k\right)\psi \in P,\;\forall k\in \left\{0,\ldots ,k_{\max}\right\},$$

while p(k)∉S if and only if

$$Q\left(k\right)\psi \notin S,\;\forall k\in \left\{0,\ldots ,k_{\max}\right\}.$$

In the following section, we assume that the constraints in the form

Q(k)θ∈P,Q(k)θ∉S

can be equivalently expressed as

$$g_{k}\left(\theta \right)=g\left(Q\left(k\right)\theta \right)\leq 0_{q}$$

for some q≥0 and for some differentiable function $g:R^{2}\to R^{q}$. In this view, the above unconstrained MLE problem can be extended as follows:

(14) $$\begin{array}{ccc} \; & \theta^{*}=\arg\min_{\theta }\lambda \left(\theta \right) & \\ \; & \mathrm{subject}\;\mathrm{to} & \\ \; & g_{k}\left(\theta \right)\leq 0_{q},\;\forall k\in \left\{0,\ldots ,k_{\max}\right\}. \end{array}$$

### 2.4. Computational Approach to Solve MLE Problems

Notice that, as remarked in [38], the MLE for bearings-only target motion analysis does not admit a closed-from solution and must be implemented iteratively, considering an initialization close to the true solution to avoid divergence. In particular, we point out that the above MLE minimization problems (both in the unconstrained and constrained fashion) are not, in general, convex, thus calling for approximated solution schemes that typically aim to find a good local optimum. In this paper, we resort to the MIDACO-SOLVER optimization software, which implements an extension of the evolutionary ant colony optimization meta-heuristic [39] and which has been developed especially for highly nonlinear real-world applications. See [40] or [41] for a focus of the performance of MIDACO software with respect to the state of the art. Notably, MIDACO-SOLVER allows one to evaluate the satisfaction of the constraints and the objective function from an algorithmic standpoint, thus allowing one to also tackle the problems that are not easily expressed in a closed form nor easily solved by traditional solvers. Note that the suggested strategy is independent of a particular solver, but the nonconvex nature of the optimization problem suggests an evolutionary approach, such as genetic algorithms [42].

### 2.5. *CRLB* of the Estimate in the Unconstrained Case

Let us now discuss a useful metric that represents a lower bound on the covariance matrix associated to the MLE estimation process. Specifically, in this subsection, we consider the unconstrained case, while the constrained one is discussed in the next subsection.

In particular, consider the problem of evaluating the CRLB for the estimated vector ψ of target parameters (see, for instance [37], p.44). In particular, it is well known that the covariance matrix is bounded by the inverse of the Fisher information matrix (FIM)*J*, i.e., it holds that

$$E\left\{\left(\theta^{*}-\psi \right){\left(\theta^{*}-\psi \right)}^{T}\right\}\geq J^{-1}\left(\bar{\psi }\right)={\mathrm{CRLB}}^{\mathrm{unconstrained}},$$

where

$$J\left(\bar{\psi }\right)=E\left\{\left[\nabla_{\theta }\lambda \left(\theta \right)\right]{\left[\nabla_{\theta }\lambda \left(\theta \right)\right]}^{T}\right\}{|}_{\theta =\bar{\psi }}.$$

Note that the expectations in the above equation are taken with respect to p(θ); moreover, the derivatives in J(·) are evaluated at the true value of ψ (i.e., $\bar{\psi }=\psi$) or, alternatively, at the estimated value $\theta^{*}$ (i.e., $\bar{\psi }=\theta^{*}$) if the true value is unknown. Let us now present a more direct expression of the FIM. To this end, let us express the gradient of λ(θ) with respect to θ as [3]

(15) $$\begin{array}{cc} \nabla_{\theta }\lambda \left(\theta \right) & =-\sum_{k=1}^{k_{\max}}\frac{1}{\sigma^{2}}\left(z\left(k\right)-h\left(\theta ,k\right)\right)\nabla_{\theta }h\left(\theta ,k\right); \end{array}$$

then, we have that [3]

(16) $$\begin{array}{cc} E\left\{\left[\nabla_{\theta }\lambda \left(\theta \right)\right]{\left[\nabla_{\theta }\lambda \left(\theta \right)\right]}^{T}\right\}{|}_{\psi }\; & \;=\;\frac{1}{\sigma^{4}}\sum_{k=1}^{k_{\max}}E\left\{{\left(z\left(k\right)-h\left(\psi ,k\right)\right)}^{2}\right\}\;\nabla_{x}h\left(\psi ,k\right)\;\nabla_{\psi }h{\left(\psi ,k\right)}^{T}\;\\ \; & =\frac{1}{\sigma^{2}}\sum_{k=1}^{k_{\max}}\nabla_{\psi }h\left(\psi ,k\right)\nabla_{\psi }h{\left(\psi ,k\right)}^{T}, \end{array}$$

where in the first equation, we used the fact that the measures are independent in order to neglect mixed terms, while the last equation follows from the consideration that

$$E\left\{{\left(z\left(k\right)-h\left(\psi ,k\right)\right)}^{2}\right\}=E\left\{w{\left(k\right)}^{2}\right\}=\sigma^{2}.$$

Notice that, in the Appendix A, we provide the analytical expression of the entries of $\nabla_{\psi }h\left(\psi ,k\right)$.

### 2.6. *CRLB* for Constrained MLE

As demonstrated in [33] (see also [34]), assuming $\theta \in R^{n}$, when the MLE problem has an equality constraint in the form

$$f\left(\theta \right)=0_{q},\;f:R^{n}\to R^{q},$$

the CRLB can be computed starting from the unconstrained case, according to the following equation

$${\mathrm{CRLB}}^{\mathrm{constrained}}=J^{-1}\left(\bar{\psi }\right)-J^{-1}\left(\bar{\psi }\right)F\left(\bar{\psi }\right){\left(F^{T}\left(\bar{\psi }\right)J^{-1}\left(\bar{\psi }\right)F\left(\bar{\psi }\right)\right)}^{-1}F^{T}\left(\bar{\psi }\right)J^{-1}\left(\bar{\psi }\right),$$

where $F(\bar{\psi })$ is the n×q Jacobian matrix of the constraint function f, evaluated at $\bar{\psi }$, i.e.,

$$F\left(\bar{\psi }\right)=\nabla_{\theta }f^{T}\left(\theta \right){|}_{\theta =\bar{\psi }}.$$

Let us now extend this method to the case of inequality constraints. To this end, consider a constraint in the form of

$$g\left(\theta \right)\leq 0_{q}$$

where $g:R^{n}\to R^{q}$ is differentiable. We point out that the inequality constraint can equivalently be expressed in the form of an equality constraint using

$$f\left(\theta \right)=\max\left\{g\left(\theta \right),0_{q}\right\}=0_{q},$$

where max is intended component-wise. Notably, the max function is globally Lipschitz (e.g., see [43]); hence, if g is differentiable everywhere, we have that f is differentiable for all $\theta \in R^{n}\backslash \Omega$, where Ω is a zero-measure set in the form

$$\Omega =\left\{\theta \in R^{n}\;|\;g_{i}\left(\theta \right)=0,\mathrm{for}\;\mathrm{some}\;i\in \left\{1,\ldots ,q\right\}\right\}.$$

In this view, a natural extension is to resort to the *generalized Jacobian* of f. Specifically, it follows from [35] that the generalized Jacobian $F_{G}\left(\theta \right)$ of f(θ) is defined as

(17) $$F_{G}\left(\theta \right)\;=\;\bar{\mathrm{co}}\left\{\lim_{i\to \infty }F\left(\theta_{i}\right)\;:\theta_{i}\to \theta ,\;\theta_{i}\notin \Omega \right\},$$

with $\bar{\mathrm{co}}$ being the convex closure, $F\left(\theta_{i}\right)\in R^{n\times m}$ the classical Jacobian whenever it exists, and Ω the set of measure zero where $F(\theta_{i})$ is not defined. In other words, $F_{G}\left(\theta \right)$ is in general set-valued at the points where the Jacobian is not defined and $F_{G}\left(\theta \right)=\left\{F\left(\theta \right)\right\}$ when the Jacobian is defined. Consequently, in the case of inequality constraints, the CRLB is also, in general, set-valued, and it holds that

$${\mathrm{CRLB}}^{\mathrm{constrained}}=\left\{J^{-1}\left(\bar{\psi }\right)-J^{-1}\left(\bar{\psi }\right)F{\left(F^{T}J^{-1}\left(\bar{\psi }\right)F\right)}^{-1}F^{T}J^{-1}\left(\bar{\psi }\right)|\;F\in F_{G}\left(\bar{\psi }\right)\right\}.$$

Notice that, in the event that $\bar{\psi }$ coincides with a point in the zero-measure set Ω, any metric based on the CRLB (e.g., variance for a specific parameter, norm of the matrix, etc.) is computed considering the worst case over the elements in the set ${\mathrm{CRLB}}^{\mathrm{constrained}}$.

### 2.7. Ownship Trajectory Selection Based on Artificial Potential Fields

The availability of additional information (i.e., knowledge on P and S) can be leveraged by the ownship in order to select a trajectory that allows one to improve the accuracy of the estimate, i.e., by moving along a direction that mediates between the attempt to get closer to P and the will to avoid S. In this paper, we borrow some of the key concepts of the so-called artificial potential fields (APF) technique [31,32] in order to accomplish this task. Within the APF approach, a robot has to navigate in a space toward a goal while avoiding one or more obstacles; this is achieved by associating a repulsive potential field to each obstacle and an attractive potential field to the goal so that, depending on the robot’s position, the robot moves in the direction of the force that corresponds to the antigradient of the overall potential field.

For the application at hand in this paper, we consider a repulsive potential field to be associated with each set $Y_{i}$ that the target is assumed not to cross and an attractive potential field associated with each set $X_{i}$ where the target is assumed to be confined in. For simplicity, assume the ownship is initially in the origin, considering some fixed frame of reference.

In detail, referring to $y_{i}$ and $x_{i}$ as the center of mass of $Y_{i}$ and $X_{i}$, respectively, the potential field at a point $p\in R^{2}$ is the superposition of a contribution

$$-\frac{1}{2}\alpha_{i}{\left(x_{i}-p\right)}^{T}\left(x_{i}-p\right)$$

for each set $X_{i}$ and a contribution

$$\frac{1}{2}\beta_{i}{\left(y_{i}-p\right)}^{T}\left(y_{i}-p\right)$$

for each set $Y_{i}$, i.e.,

$$U\left(p\right)=-\frac{1}{2}\sum_{i=1}^{m}\alpha_{i}{\left(x_{i}-p\right)}^{T}\left(x_{i}-p\right)+\frac{1}{2}\sum_{i=1}^{r}\beta_{i}{\left(y_{i}-p\right)}^{T}\left(y_{i}-p\right),$$

from which the corresponding force at the origin is

$$f\left(0_{2}\right)=-\nabla_{p}U\left(p\right){|}_{p=0_{2}}=\sum_{i=1}^{m}\alpha_{i}x_{i}-\sum_{i=1}^{m}\beta_{i}y_{i},$$

and we note that the force is the composition of terms that are attracting toward the points $x_{i}$ and terms that are repulsive from the points $y_{i}$.

Notice that, in this paper, we chose coefficients $\beta_{i}$ that are proportional to the area of the corresponding set $Y_{i}$ (i.e., the larger $Y_{i}$ is, the more the force is repulsive); conversely, we chose coefficients $\alpha_{i}$ that are inversely proportional to the area of the corresponding set $Y_{i}$ (i.e., the smaller $X_{i}$ is, the more the force is attractive).

#### Ownship Trajectory

Based on the resulting force $f(0_{2})$ at the origin, the ownship computes the angle γ between the vector ${\left[\begin{array}{cc} 0 & 1 \end{array}\right]}^{T}$ and $f(0_{2})$ and moves according to

(18) $$\left[\begin{array}{c} x_{o}\left(kT\right)\\ y_{o}\left(kT\right) \end{array}\right]=\left[\begin{array}{cc} \cos(\gamma ) & -\sin(\gamma )\\ \sin(\gamma ) & \cos(\gamma ) \end{array}\right]\left[\begin{array}{c} {\bar{\dot{x}}}_{o}kT\\ A\sin(\omega x_{o}\left(kT\right)) \end{array}\right],$$

i.e., the ownship moves of linear motion (and constant velocity ${\bar{\dot{x}}}_{o}$) along the direction of $f(0_{2})$ while performing sinusoidal motion around such a direction. Notably, Equation (18) can be rearranged as

(19) $$\left[\begin{array}{c} x_{o}\left(kT\right)\\ y_{o}\left(kT\right) \end{array}\right]=\left[\begin{array}{c} \cos\left(\gamma \right){\bar{\dot{x}}}_{o}kT-A\sin\left(\gamma \right)\sin\left(\omega x_{o}\left(kT\right)\right)\\ \sin\left(\gamma \right){\bar{\dot{x}}}_{o}kT+A\cos\left(\gamma \right)\sin\left(\omega x_{o}\left(kT\right)\right) \end{array}\right].$$

**Figure 1:**
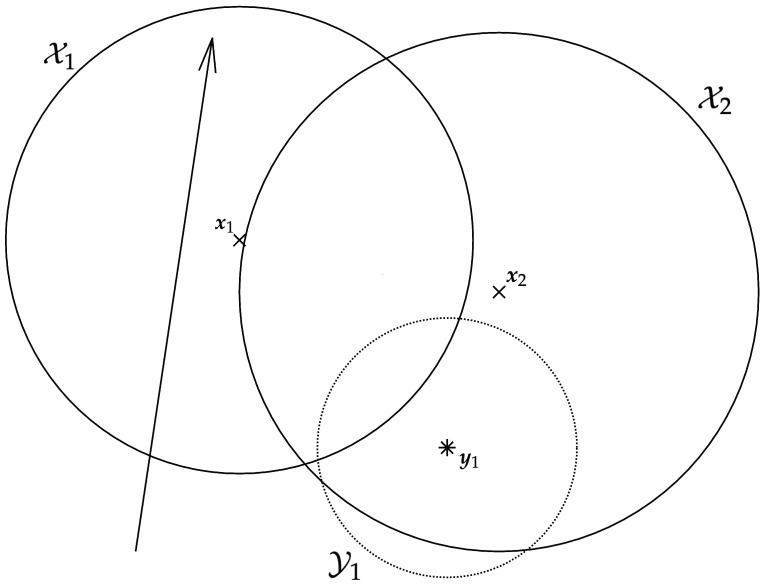
Example of the proposed approach for selecting the ownship’s trajectory. In this example, we consider two sets, $X_{1}$ and $X_{2}$, and one setm $Y_{1}$, represented by the circles shown with a solid line and by a dotted line, respectively. The points $x_{1}$ and $x_{2}$ and the point $y_{1}$ (i.e., the centers of the circles) are shown with an x mark and with a cross, respectively. In this example, we choose $\alpha_{1}$ and $\alpha_{2}$ equal to the area of $X_{1}$ and $X_{2}$, respectively, while $\beta_{1}$ is the reciprocal of the area of $Y_{1}$. The resulting direction for the ownship is shown with an arrow (the initial position for the ownship is given by the starting endpoint of the arrow).

Figure 1 provides an example of the above procedure for the selection of $f(0_{2})$.

## 3. Experimental Analysis

**Figure 2:**
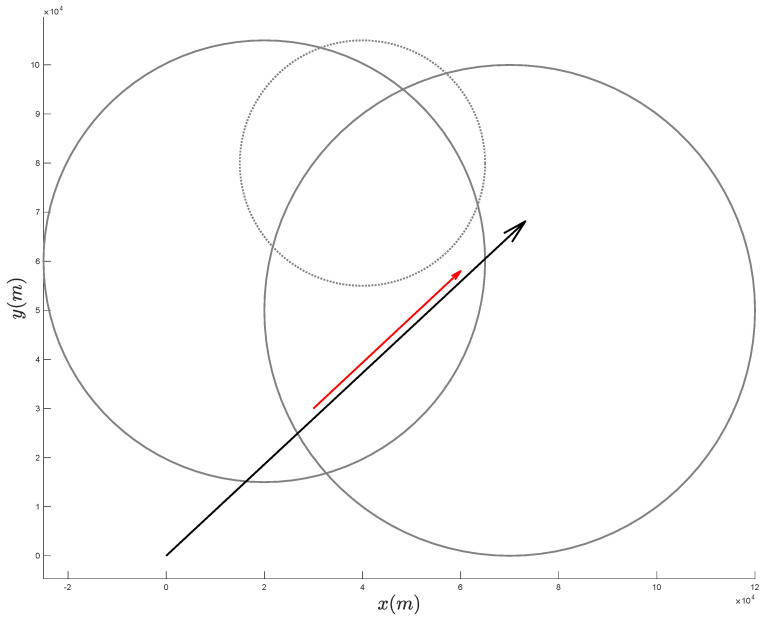
Scenario considered in the experimental analysis. We assume additional information is available, in that the target’s trajectory is known to be confined in the intersection of the two solid circles and to lie outside the dotted circle. The red (shorter) arrow represents the target’s trajectory. The APF direction is shown with a black (longer) arrow.

In order to experimentally demonstrate the effectiveness of the proposed approach, we consider the scenario depicted in Figure 2, where the target has an initial position $x_{t0}=y_{t0}=3\times 10^{4}\;m$ and moves with constant velocity having components ${\dot{x}}_{t0}=8.333\;m/s$ and ${\dot{y}}_{t0}=7.778\;m/s$, while the acceleration is ${\ddot{x}}_{t0}={\ddot{y}}_{t0}=0\;m/s^{2}$.

Notice that we assume the additional information is available to the ownship regarding the target’s trajectory. Specifically, we assume the target’s trajectory lies in the intersection of the circles $X_{1}$ and $X_{2}$ and outside of the circle $Y_{1}$; the centers $x_{1},x_{2},y_{1}$ and the radii $\rho_{X_{1}},\rho_{X_{2}}$ and $\rho_{Y_{1}}$ of such circles are reported in Table 1.

In our simulations, we consider a sampling time $T=2\;\begin{array}{c} s \end{array}$, a time horizon of $3600\;\begin{array}{c} s \end{array}$ and noise variance set to $\sigma =0.5^{\circ }$.

As for the ownship, we consider four operational scenarios:

- No information: the ownship does not rely on the additional information and selects the trajectory in Equation (19) with $\gamma =0\begin{array}{c} \mathrm{rad} \end{array}$.
- Unconstrained, APF direction: the ownship does not rely on the additional information for the computations but selects the trajectory according to the proposed APF approach; in other words, it selects the trajectory in Equation (19) with $\gamma =0.749\begin{array}{c} \mathrm{rad} \end{array}$.
- Constrained, no APF direction: the ownship relies on the additional information for the computations but selects the trajectory in Equation (19) with $\gamma =0\begin{array}{c} \mathrm{rad} \end{array}$.
- Proposed Approach: the ownship follows the trajectory in Equation (19) with $\gamma =0.749\begin{array}{c} \mathrm{rad} \end{array}$ and, further to that, actively relies on the additional information during the computation of the MLE solution, of the experimental covariance matrix and CRLB.

In all cases, we select the following parameters for the ownship: $x_{o}\left(0\right)=y_{o}\left(0\right)=0\begin{array}{c} m \end{array}$, ${\dot{x}}_{o}=7.778\begin{array}{c} m/s \end{array}$, $\omega =2.5\times 10^{-4}\begin{array}{c} rad/s \end{array}$ and $A=5\times 10^{3}\begin{array}{c} m \end{array}$.

The computation of the MLE solution with MIDACO-SOLVER was conducted on an Intel^®^ i7 quad-core @ 2.27 GHz. For each execution of MIDACO-SOLVER, we set the number of evaluated solutions to $10^{6}$. All other MIDACO-SOLVER parameters were used by their default values, which especially means that a feasibility accuracy of 0.001 was used for all individual constraints. In all cases, we compute the MLE solution with MIDACO-SOLVER, and we consider 100 MLE solutions for each operational scenario.

Table 2 reports the results obtained for the four operational scenarios, considering both the best solution found (in terms of the objective function of the MLE) over the 100 runs and the average of the solutions found. For each solution reported in the table, we use, as a measure of quality of the estimate the relative position and relative velocity, defined as follows:

(20) $$\mathrm{rel}.\;\mathrm{pos}.\;\mathrm{err}.\;={∥\left[\begin{array}{c} \frac{x_{t0}-x_{t0}^{*}}{x_{t0}}\\ \frac{y_{t0}-y_{t0}^{*}}{y_{t0}} \end{array}\right]∥}_{2}$$

(21) $$\mathrm{rel}.\;\mathrm{vel}.\;\mathrm{err}.\;={∥\left[\begin{array}{c} \frac{{\dot{x}}_{t0}-{\dot{x}}_{t0}^{*}}{{\dot{x}}_{t0}}\\ \frac{{\dot{y}}_{t0}-{\dot{y}}_{t0}^{*}}{{\dot{y}}_{t0}} \end{array}\right]∥}_{2}$$

while, since the target moves of with zero acceleration, we consider the absolute acceleration error

(22) $$\mathrm{abs}.\;\mathrm{acc}.\;\mathrm{err}.\;={∥\left[\begin{array}{c} {\ddot{x}}_{t}^{*}\\ {\ddot{y}}_{t}^{*} \end{array}\right]∥}_{2}$$

where the asterisk superscript is used for the estimated parameters. According to the table, the best solution found in the first and proposed operative scenarios is comparable and exhibits small error, while the error is larger for the third operational scenario. Conversely, within the second operational scenario, the best solution also found is not satisfactory, due to large relative velocity error. The situation is radically different considering the average of the found solutions; in fact, we observe that while the proposed approach shows limited error, the first and second operational conditions are characterized by erroneous average solutions (especially for what concerns the estimate of the target’s initial velocity), while the third one exhibits a limited but significant degradation. This suggests that, while the proposed approach consistently finds a good solution over the different trials, the other methods may become trapped by worst local minima.

**Figure 3:**
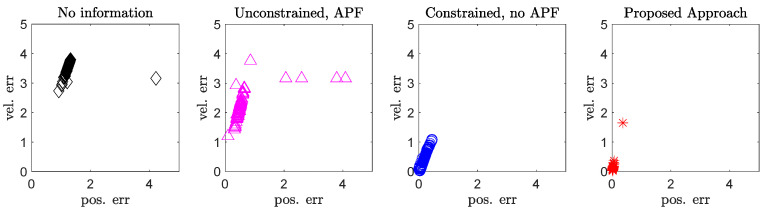
Ensemble view of the (relative) position and velocity error values over 100 runs (i.e., see Equations (20) and (21)), considering the four operative scenarios.

This intuition is supported by Figure 3, where we show the distribution of the position and velocity error values over the 100 trials. According to the figure, it can be noted that the first two scenarios have, overall, worse performance than the second and third scenario. Moreover, while outliers in the first two operative scenarios assume remarkably large values, in the latter two scenarios the outliers exhibit only a limited increase.

In order to further analyze the different operational scenarios, Table 3 reports the norm of the covariance matrix obtained experimentally from the 100 trials, the experimental covariance limited to the solutions with errors within the 50th percentile, and the norm of the CRLB. In other words, we experimentally evaluate the covariance by executing 100 instances of MIDACO-SOLVER with random initial choice for the parameters and then taking the covariance of the 100 (or less, when only the 50th percentile is considered), possibly different, solutions obtained via MIDACO-SOLVER.

Notably, for the proposed approach, due to the presence of inequality constraints, the CRLB is in general set-valued and, following a worst-case philosophy, when the set is not a singleton, we consider

(23) $$\max_{C\in {\mathrm{CRLB}}^{\mathrm{constrained}}}{∥C∥}_{2}.$$

According to the table, the magnitude of the norm of the experimental covariance matrices associated with the proposed approach is three orders of magnitude smaller than those corresponding to the first and second operational scenarios, while it is two orders of magnitude smaller than the covariance associated to the third operational scenario. Moreover, the norm of the CRLB covariance matrices is two orders of magnitude smaller than the one obtained for the first and second operational scenarios, while it is comparable to the one associated with the third operational scenario. Moreover, we observe that, while in the other cases the experimental covariance is between three and four orders of magnitude larger than the CRLB, for the proposed approach, the experimental covariance is just two orders of magnitude larger than the CRLB. Notably, the discrepancy experienced between the experimental covariance computed over 100 trials and the CRLB one is due to the structure of the problem at hand. In fact, the MLE problem being solved amounts to a nonconvex, nonlinear programming problem and is thus characterized by the presence of local minima. Since we are adopting an approximated solver for finding a solution, the large experimental covariance is explained by the reach of different local minima across the different trials. In fact, while analyzing the covariance restricted to solutions with errors within the 50th percentile, we observe a significant drop with respect to the covariance over all trials; in particular, we observe a reduction of two orders of magnitude for the first two operational scenarios and one order of magnitude for the other two; moreover, also in this case, the proposed approach shows a two orders of magnitude reduction of the covariance with respect to the others.

Let us now discuss the computation times, which are collected in Table 4. According to the table, we observe that the main differences arise between the unconstrained and the constrained cases, the latter requiring a computational time that is, on average, about 46% larger than the unconstrained case, while the standard deviation is sensibly larger, being about 6.92 times the one obtained in the unconstrained case.

Overall, the above results suggest that, while the implementation of the constrained MLE computation without considering the APF direction has a positive effect on the estimate, the APF direction alone has no particular benefit without constraining the MLE based on the available information. Instead, when such innovations are combined, the MLE error, the experimental covariance and the CRLB are greatly reduced. Notably, since the proposed approach amounts to a constrained problem, the price to pay is a noticeable but limited increase in the computation times.

**Figure 4:**
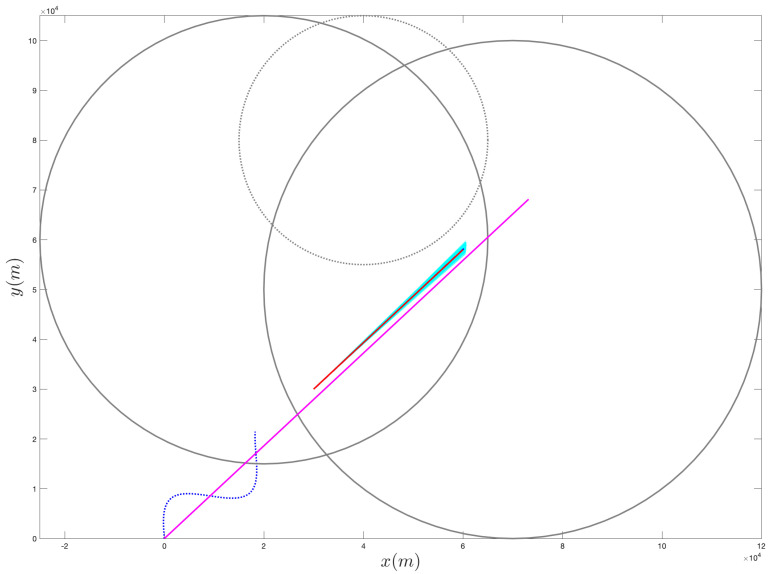
Sampling of 100 trajectories (in cyan) based on the average solution found via the proposed approach and on the experimental covariance matrix.

**Figure 5:**
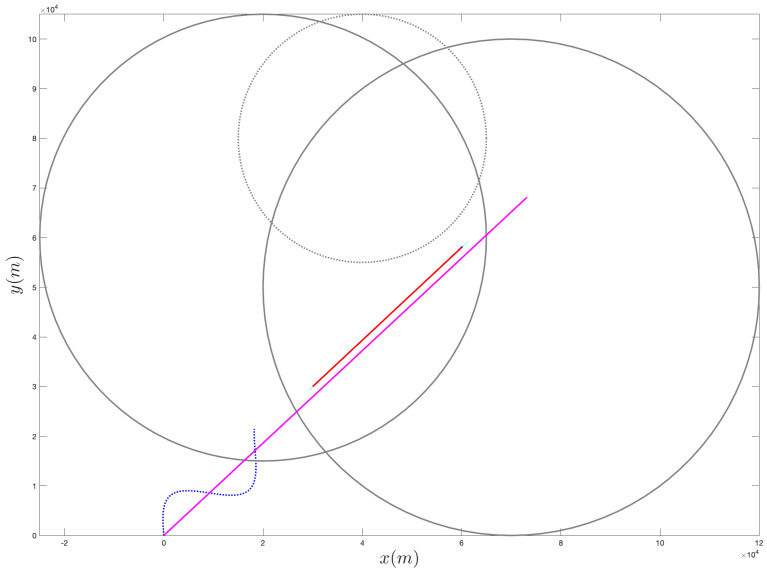
Sampling of 100 trajectories (cyan) based on the average solution found via the proposed approach and on the CRLB covariance matrix.

**Figure 6:**
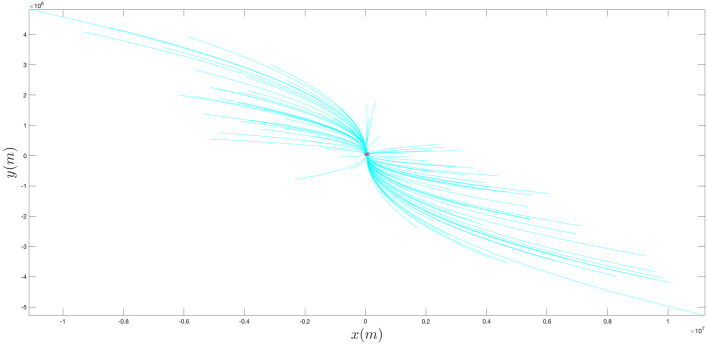
Sampling of 100 trajectories (cyan) based on the best solution found via the unconstrained, APF direction approach and on the CRLB covariance matrix.

**Figure 7:**
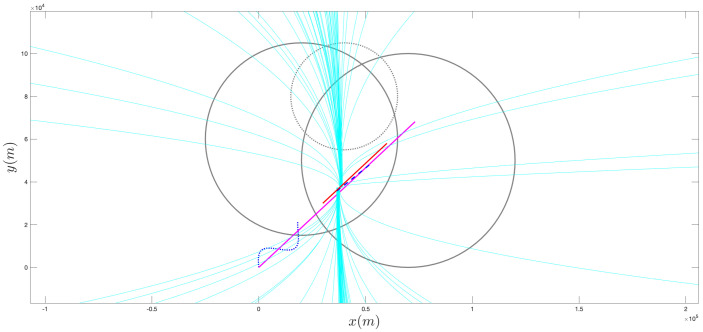
Zoomed version of a portion of Figure 6.

**Figure 8:**
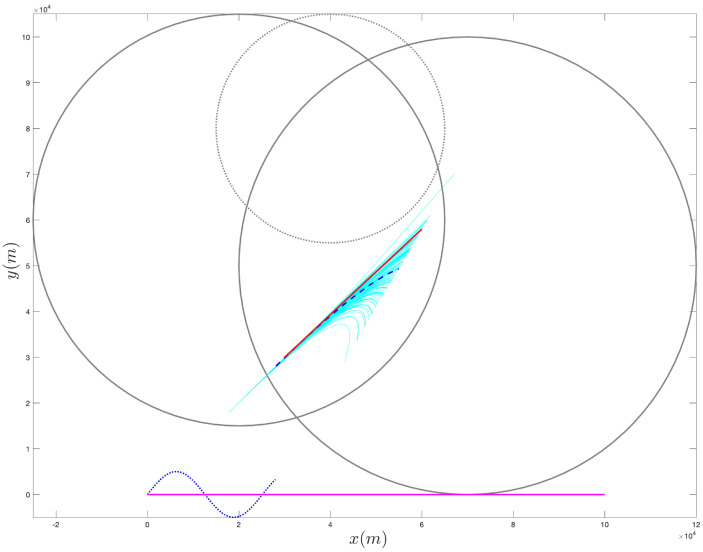
Sampling of 100 trajectories (cyan) based on the best solution found via for the constrained case, but without relying on the APF, and on the experimental covariance matrix.

**Figure 9:**
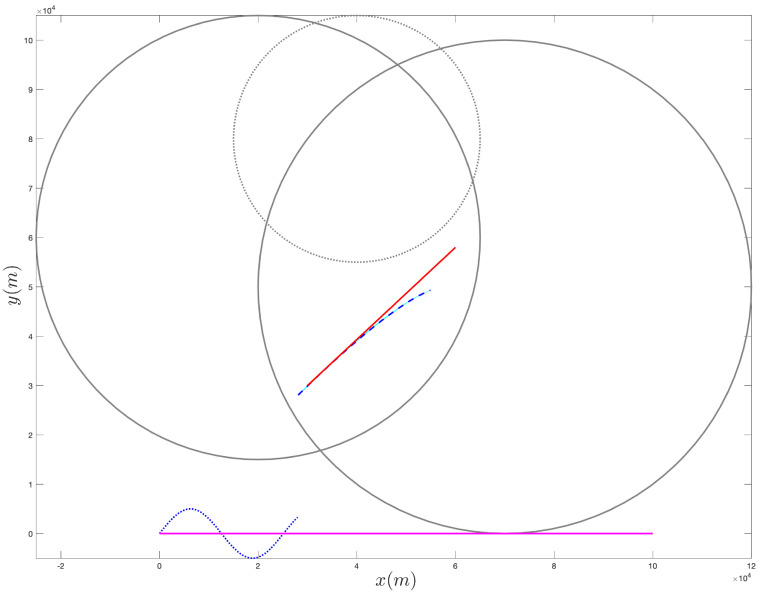
Sampling of 100 trajectories (cyan) based on the best solution found via for the constrained case, but without relying on the APF, and on the CRLB covariance matrix.

Figure 4, Figure 5, Figure 6, Figure 7, Figure 8 and Figure 9 provide an at-a-glance view of the performance gap when the additional information is actively relied upon. Specifically, Figure 4 and Figure 5 show the results of the proposed operational scenario considering the average MLE solution over the 100 trials (blue dashed line), along with 100 trajectories (in cyan) obtained by sampling the parameters from a Gaussian distribution with mean given by the average MLE result and covariance corresponding to the experimental covariance matrix or the CRLB, respectively (the ownship’s trajectory is shown via the blue sinusoidal dashed curve). Conversely, Figure 6 shows the results obtained considering the case where the APF direction is used but the additional information is not relied upon during the MLE computation; specifically, the figure considers the best solution found and the CRLB matrix. Notably, in the latter case, the large covariance yields sampled trajectories that are characterized by remarkably large error, while the proposed approach (both considering the experimental and CRLB covariance matrices) yields significantly better results. Figure 7 shows a zoomed version of Figure 6; according to the figure, we observe that, while the APF approach without using the additional information in the MLE computations is characterized by an initial position that is relatively accurate, the velocities and accelerations are characterized by highly variable and erroneous values, resulting in the inaccurate trajectories. Finally, Figure 8 and Figure 9 show the results obtained considering the case where the APF direction is not used but the additional information is relied upon during the MLE computation; specifically, Figure 8 consider the best solution found and the experimental covariance matrix, while Figure 8 considers the best solution found and the CRLB matrix; in this case, the trajectory is characterized by an intermediate error and is outperformed by the proposed approach.

To conclude the section, we compare the proposed approach against other methods in the literature. Specifically, our proposed comparison is based on two macro-steps: (i) the estimation of the target’s trajectory for $k\in \{0,\ldots k_{\max}\}$ via other approaches in the literature and (ii) the comparison with the trajectory obtained based on the estimation of the parameter vector ψ via the proposed approach. In particular, we estimate the target’s trajectory by resorting to the following four algorithms:

- a standard extended Kalman filter (EKF) (e.g., see [44] and references therein), where we approximate the nonlinear output function h(·) by its Jacobian matrix at each time instant;
- the pseudolinear Kalman filter (PL-KF) [45], where the nonlinear and noisy output $z\left(k\right)=\begin{array}{c} \mathrm{atan}2(y_{t}\left(k\right)-y_{o}\left(k\right),x_{t}\left(k\right)-x_{o}\left(k\right)) \end{array}+w\left(k\right)$ is approximated by
  $$\tilde{z}\left(k\right)=\left[\begin{array}{c} \sin(z(k))\\ -\cos(z(k)) \end{array}\right]\underset{M}{\underset{︸}{\left[\begin{array}{cccccc} 1 & 0 & 0 & 0 & 0 & 0\\ 0 & 1 & 0 & 0 & 0 & 0 \end{array}\right]}}{\hat{x}}_{k|k}+\eta \left(k\right),$$
  where ${\hat{x}}_{k|k}$ is the vector collecting the filtered states (i.e., the stack of the positions, velocities and accelerations) of the target at time *k* and η(k) is a pseudolinear noise in the form
  $$\eta \left(k\right)∼N\left(0,R_{k}\right),$$
  with
  $$R_{k}\approx {∥{\hat{d}}_{k|k-1}∥}^{2}\sigma^{2}$$
  and
  $${\hat{d}}_{k|k-1}=M{\hat{x}}_{k|k-1}-\left[\begin{array}{c} x_{o}\left(k\right)\\ y_{o}\left(k\right) \end{array}\right],$$
  ${\hat{x}}_{k|k-1}$ being the vector collecting the prediction of the target’s states at time *k*;
- a statistical linearization extended Kalman filter (SL-EKF) [46] where, instead of the Jacobian of the output function h(·), we approximate the nonlinear measurement function y(k)=h(x,k)+w(k) via the linear approximation $H^{*}x$, with
  $$H^{*}=\underset{H\in R^{2\times 6}}{\arg\min}{∥y\left(k\right)-H{\hat{x}}_{k|k}∥}^{2}.$$
- the shifted Rayleigh filter (SRF) [47], where z(k) is approximated by
  $$z\left(k\right)\approx \Pi \left(Mx(k)+u(k)+\omega (k)\right),$$
  where Π(r)=r/∥r∥, $u\left(k\right)=\left[\begin{array}{cc} x_{o}\left(k\right) & y_{o}\left(k\right) \end{array}\right]$ and
  $$\omega \left(k\right)∼N\left(0_{2},\sigma^{2}E\left[{∥Mx\left(k\right)+u\left(k\right)∥}^{2}\;|\;z\left(1\right),\ldots ,z\left(k\right)\right]I_{2}\right).$$

Conversely, within the proposed approach, we estimate the parameter vector

$$\psi ={\left[\begin{array}{cccccc} x_{t0} & y_{t0} & {\dot{x}}_{t0} & {\dot{y}}_{t0} & {\ddot{x}}_{t} & {\ddot{y}}_{t} \end{array}\right]}^{T}$$

via the proposed constrained MLE formulation, and we compute the trajectory of the target as follows

x(k)=Q(k)ψ,

where

$$x\left(k\right)={\left[\begin{array}{cccccc} x_{t}\left(k\right) & y_{t}\left(k\right) & {\dot{x}}_{t}\left(k\right) & {\dot{y}}_{t}\left(k\right) & {\ddot{x}}_{t}\left(k\right) & {\ddot{y}}_{t}\left(k\right) \end{array}\right]}^{T},$$

and

$$Q\left(k\right)=\left[\begin{array}{ccc} I_{2} & kTI_{2} & \frac{1}{2}k^{2}T^{2}I_{2}\\ 0_{2\times 2} & I_{2} & kTI_{2}\\ 0_{2\times 2} & 0_{2\times 2} & I_{2} \end{array}\right].$$

Regarding the initial condition for the algorithms compared against the proposed approach, we consider three different scenarios, with a decreasing degree of uncertainty:

(1) a scenario where the average initial condition ${\hat{x}}_{0|0}$ is drawn from a zero-mean Gaussian variable with standard deviation equal to ψ, while the initial covariance $\Sigma_{0|0}$ is equal to the square of ψ, i.e.,
  $${\hat{x}}_{0|0}∼N\left(0_{6},\begin{array}{c} \mathrm{diag} \end{array}{\left(\psi \right)}^{2}\right),\;\Sigma_{0|0}=\begin{array}{c} \mathrm{diag} \end{array}{\left(\psi \right)}^{2};$$
(2) a scenario where the average initial condition is drawn from a Gaussian variable with a mean equal to ψ and standard deviation equal to ψ, while the initial covariance is equal to the square of ψ, i.e.,
  $${\hat{x}}_{0|0}∼N\left(\psi ,\begin{array}{c} \mathrm{diag} \end{array}{\left(\psi \right)}^{2}\right),\;\Sigma_{0|0}=\begin{array}{c} \mathrm{diag} \end{array}{\left(\psi \right)}^{2}$$
(3) a scenario where the average initial condition is exactly ψ, while the initial covariance is equal to the square of ψ, i.e.,
  $${\hat{x}}_{0|0}=\psi ,\;\Sigma_{0|0}=\begin{array}{c} \mathrm{diag} \end{array}{\left(\psi \right)}^{2}.$$

**Figure 10:**
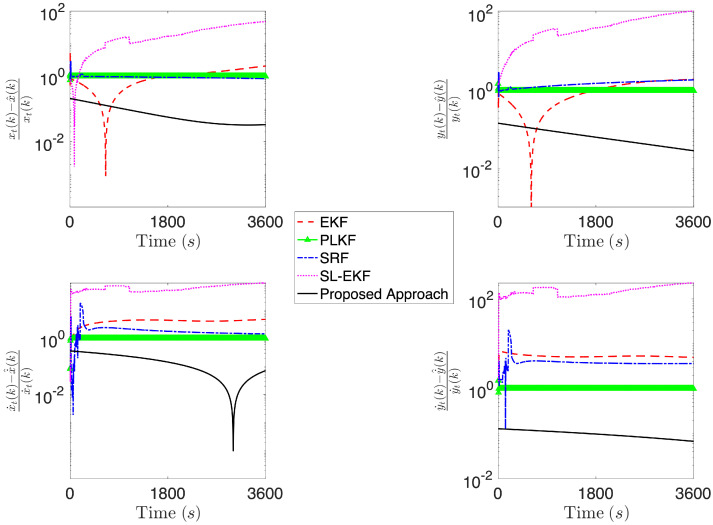
Comparison of the proposed approach against EKF, PLKF, SRF and SL-EKF, considering a scenario where the methods compared with the proposed one are initialized with ${\hat{x}}_{0|0}∼N\left(0_{6},\begin{array}{c} \mathrm{diag} \end{array}{\left(\psi \right)}^{2}\right)$ and $\Sigma_{0|0}=\begin{array}{c} \mathrm{diag} \end{array}{\left(\psi \right)}^{2}$.

**Figure 11:**
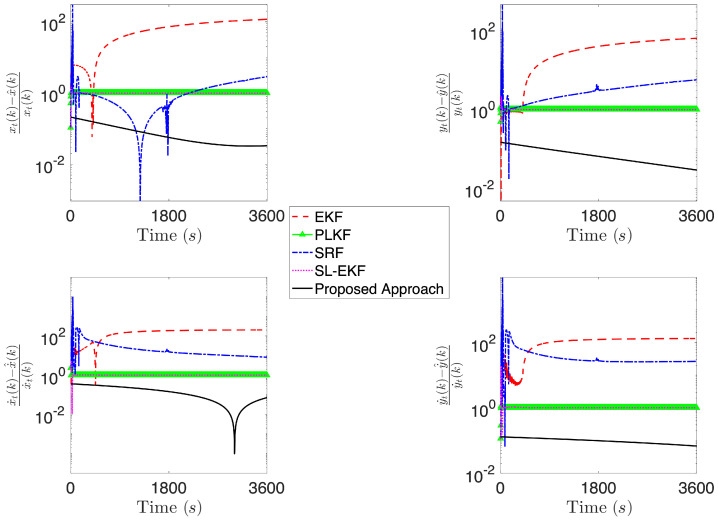
Comparison of the proposed approach against EKF, PLKF, SRF and SL-EKF, considering a scenario where the methods compared with the proposed one are initialized with ${\hat{x}}_{0|0}∼N\left(\psi ,\begin{array}{c} \mathrm{diag} \end{array}{\left(\psi \right)}^{2}\right)$ and $\Sigma_{0|0}=\begin{array}{c} \mathrm{diag} \end{array}{\left(\psi \right)}^{2}$.

**Figure 12:**
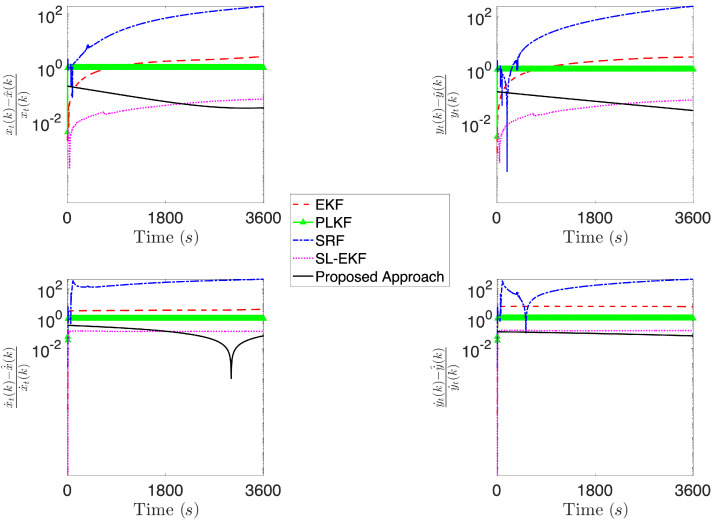
Comparison of the proposed approach against EKF, PLKF, SRF and SL-EKF, considering a scenario where the methods compared with the proposed one are initialized with ${\hat{x}}_{0|0}=\psi$ and $\Sigma_{0|0}=\begin{array}{c} \mathrm{diag} \end{array}{\left(\psi \right)}^{2}$.

Figure 10, Figure 11 and Figure 12 report the results of the comparison, where the four aforementioned approaches are compared against the average MLE solution over 100 trials obtained via the proposed methodology, considering the same simulation setting as in the fourth operational scenario described above. Specifically, we report the relative position and velocity errors between the real target’s trajectory at time *k* and the estimated one, i.e.,

$$\frac{x_{t}\left(k\right)-\hat{x}\left(k\right)}{x_{t}\left(k\right)},\;\frac{y_{t}\left(k\right)-\hat{y}\left(k\right)}{y_{t}\left(k\right)},\;\frac{{\dot{x}}_{t}\left(k\right)-\hat{\dot{x}}\left(k\right)}{{\dot{x}}_{t}\left(k\right)},\;\frac{{\dot{y}}_{t}\left(k\right)-\hat{\dot{y}}\left(k\right)}{{\dot{y}}_{t}\left(k\right)},$$

where we denote by $\hat{x}\left(k\right),\hat{y}\left(k\right),\hat{\dot{x}}\left(k\right)$ and $\hat{\dot{y}}\left(k\right)$, the estimate of the target’s positions and velocities obtained according to the generic technique being compared. According to Figure 10, the proposed approach achieves an error that is at least two orders of magnitude less than the other approaches. Notably, while moving to a scenario where more information on the initial condition is available for the four approaches used in the comparison, the gap with PLKF and SL-EKF reduces to about one order of magnitude. Finally, when we compare the initial condition for the methods against the proposed one, which is assumed to have a mean that corresponds to the actual vector of parameter being estimated, we observe that the proposed approach is comparable with SL-EKF, while being in general better than the others.

Overall, the proposed comparison contributes to experimentally demonstrating the effectiveness of the proposed approach with respect to the literature.

## 4. Conclusions and Future Work

This paper presents a batch strategy to estimate the parameters describing the trajectory of a target, based on a moving ownship able to measure bearings. In particular, the proposed methodology allows one to incorporate additional information (e.g., obtained via intelligence) such as knowledge of the fact that the target’s trajectory is contained in the intersection of some sets or the fact it lies outside the union of other sets. The approach is formally characterized by providing a constrained MLE formulation and by extending the definition of the CRLB matrix to the case of MLE problems with inequality constraints, relying on the concept of generalized Jacobian matrix. Moreover, based on the additional information, the ownship motion is chosen by mimicking the Artificial Potential Fields technique that is typically used by mobile robots to aim to a goal (in this case, the region where the target is assumed to be) while avoiding obstacles (i.e., the region that is assumed not to intersect with the target’s trajectory). As a result, the proposed framework exhibits remarkably better performance, and in particular, we observe that the solution is less likely to remain stuck in unsatisfactory local minima during the MLE computation and is characterized by smaller covariance, (both considering the experimental and the CRLB ones).

Future work will be mainly devoted to extending the framework along the following research directions: (i) consider more sophisticated models for the target’s motion (e.g., nearly constant acceleration); (ii) consider dynamically changing constraints on the target’s trajectory; (iii) provide adaptive strategies for the ownship trajectory based on the, although partial, initial estimates (e.g., in order to avoid crossing the space where the target’s trajectory is contained); (iv) filter possible outliers (e.g., resorting to the approach in [3], Section 2.7); and (v) consider multiple targets.

## Figures and Tables

**Table 1:** Parameters describing the sets $X_{1}$,$X_{2}$ and $Y_{1}$ considered in the experimental analysis.

Set	Centroid	Radius
$X_{1}$	$x_{1}={\left[10^{4},5\times 10^{4}\right]}^{T}\;m$	$\rho_{X_{1}}=5\times 10^{4}\;m$
$X_{2}$	$x_{2}={\left[2\times 10^{4},6\times 10^{4}\right]}^{T}\;m$	$\rho_{X_{2}}=4.5\times 10^{4}\;m$
$Y_{1}$	$y_{1}={\left[4\times 10^{4},8\times 10^{4}\right]}^{T}\;m$	$\rho_{Y_{1}}=2.5\times 10^{4}\;m$

**Table 2:** MLE parameter estimation via MIDACO-SOLVER for the different operational scenarios.

	$x_{t0}\;m$	$y_{t0}\;m$	${\dot{x}}_{t0}\;m/s$	${\dot{y}}_{t0}\;m/s$	${\ddot{x}}_{t}\;m/s$	${\ddot{y}}_{t}\;m/s$	rel. pos. err.	rel. vel. err.	abs. acc. err.
**Ground truth**	$3.000\times 10^{4}$	$3.000\times 10^{4}$	8.333	7.778	0.000	0.000	-	-	-
**No information, average**	$3.933\times 10^{4}$	$3.848\times 10^{4}$	−5.048	−4.123	$3.437\times 10^{-3}$	$2.303\times 10^{-3}$	$4.202\times 10^{-1}$	2.218	$4.137\times 10^{-3}$
**Unconstrained, APF direction, average**	$3.796\times 10^{4}$	$3.733\times 10^{3}$	−4.714	−4.143	$4.780\times 10^{-3}$	$3.958\times 10^{-3}$	$3.605\times 10^{-1}$	2.191	$6.206\times 10^{-3}$
**Constrained, no APF direction, average**	$3.204\times 10^{4}$	$3.193\times 10^{4}$	7.161	6.884	$2.543\times 10^{-4}$	$4.987\times 10^{-5}$	$9.384\times 10^{-2}$	$1.815\times 10^{-1}$	$2.591\times 10^{-4}$
**Proposed Approach, average**	$3.040\times 10^{4}$	$3.035\times 10^{4}$	7.912	7.382	$2.652\times 10^{-4}$	$2.598\times 10^{-4}$	$1.767\times 10^{-2}$	$7.169\times 10^{-2}$	$3.713\times 10^{-4}$
**No information, best**	$3.028\times 10^{4}$	$3.024\times 10^{4}$	8.045	7.507	$1.989\times 10^{-4}$	$1.969\times 10^{-4}$	$1.243\times 10^{-2}$	$4.902\times 10^{-2}$	$2.789\times 10^{-4}$
**Unconstrained, APF direction, best**	$3.704\times 10^{4}$	$3.640\times 10^{4}$	0.686	0.618	$3.754\times 10^{-3}$	$3.560\times 10^{-3}$	$3.171\times 10^{-1}$	1.299	$5.173\times 10^{-3}$
**Constrained, no APF direction, best**	$2.811\times 10^{4}$	$2.806\times 10^{4}$	10.081	7.382	$-1.460\times 10^{-3}$	$-2.202\times 10^{-3}$	$9.023\times 10^{-2}$	$3.412\times 10^{-1}$	$2.642\times 10^{-3}$
**Proposed Approach, best**	$3.026\times 10^{4}$	$3.023\times 10^{4}$	8.070	7.531	$1.835\times 10^{-4}$	$1.821\times 10^{-4}$	$1.179\times 10^{-2}$	$4.471\times 10^{-2}$	$2.586\times 10^{-4}$

**Table 3:** Euclidean norm of the parameter estimation via MIDACO-SOLVER for the different operational scenarios.

	Experimental Covariance ${∥\cdot ∥}_{2}$	Experimental Covariance ${∥\cdot ∥}_{2}$	CRLB ${∥\cdot ∥}_{2}$
		(Solutions with Errors ≤ 50th Percentile)	
No information	$3.851\times 10^{8}$	$3.743\times 10^{6}$	$7.713\times 10^{5}$
Unconstrained, APF direction	$5.666\times 10^{8}$	$4.144\times 10^{6}$	$7.713\times 10^{5}$
Constrained, no APF direction	$3.696\times 10^{7}$	$5.410\times 10^{6}$	$1.454\times 10^{3}$
Proposed Approach	$4.784\times 10^{5}$	$6.600\times 10^{4}$	$1.873\times 10^{3}$

**Table 4:** Average and standard deviation over 100 trials of the computational time (in seconds) for the computation of the MLE solution via MIDACO-SOLVER for the different operational scenarios.

	Time (Average) s	Time (Standard Deviation) s
No information	58.790	3.235
Unconstrained, APF direction	59.124	3.049
Constrained, no APF direction	85.926	21.231
Proposed Approach	86.230	22.395

## Data Availability

The data presented in this study are available on reasonable request from the corresponding author.

## Abbreviations

The following abbreviations are used in this manuscript:

APF	Artificial potential fields
CRLB	Cramér–Rao lower bound
DOAJ	Directory of open access journals
EKF	Extended Kalman filter
FIM	Fisher information matrix
MAP	Maximum a posteriori
MDPI	Multidisciplinary Digital Publishing Institute
MIDACO-SOLVER	Mixed integer distributed ant colony optimization solver
MLE	Maximum likelihood estimation
PL-KF	Pseudo-linear Kalman filter
SL-EKF	Statistical linearization extended Kalman filter
SRF	Shifted Rayleigh filter
TMA	Target motion analysis

## Appendix A

In this appendix we provide the analytical expression of the partial derivatives of the function h(ψ,k) with respect to the target’s motion parameters. For the sake of readability let us define

(A1) $$\begin{array}{cc} \Delta_{x}\left(k\right) & =x_{t}\left(k\right)-x\left(k\right),\\ \Delta_{y}\left(k\right) & =y_{t}\left(k\right)-y\left(k\right). \end{array}$$

The partial derivatives of h(ψ,k) with respect to the target’s motion parameters are as follows

(A2) $$\begin{array}{cc} \frac{\partial h(\psi ,k)}{\partial x_{t0}} & =-\frac{\Delta_{y}\left(k\right)}{\Delta_{x}{\left(k\right)}^{2}+\Delta_{y}{\left(k\right)}^{2}}\\ \frac{\partial h(\psi ,k)}{\partial y_{t0}} & =\frac{\Delta_{x}\left(k\right)}{\Delta_{x}{\left(k\right)}^{2}+\Delta_{y}{\left(k\right)}^{2}}\\ \frac{\partial h(\psi ,k)}{\partial {\dot{x}}_{t0}} & =-kT\frac{\Delta_{y}\left(k\right)}{\Delta_{x}{\left(k\right)}^{2}+\Delta_{y}{\left(k\right)}^{2}}\\ \frac{\partial h(\psi ,k)}{\partial {\dot{y}}_{t0}} & =kT\frac{\Delta_{x}\left(k\right)}{\Delta_{x}{\left(k\right)}^{2}+\Delta_{y}{\left(k\right)}^{2}}\\ \frac{\partial h(\psi ,k)}{\partial {\ddot{x}}_{t0}} & =-\frac{1}{2}k^{2}T^{2}\frac{\Delta_{y}\left(k\right)}{\Delta_{x}{\left(k\right)}^{2}+\Delta_{y}{\left(k\right)}^{2}}\\ \frac{\partial h(\psi ,k)}{\partial {\ddot{y}}_{t0}} & =\frac{1}{2}k^{2}T^{2}\frac{\Delta_{x}\left(k\right)}{\Delta_{x}{\left(k\right)}^{2}+\Delta_{y}{\left(k\right)}^{2}}. \end{array}$$

## References

[1] Nardone S.C., Graham M.L. (1997). A closed-form solution to bearings-only target motion analysis. IEEE J. Ocean. Eng..

[2] Song T.L., Um T.Y. (1996). Practical guidance for homing missiles with bearings-only measurements. IEEE Trans. Aerosp. Electron. Syst..

[3] Farina A. (1999). Target tracking with bearings–only measurements. Signal Process..

[4] Oh R., Song T.L., Choi J.W. (2020). Batch Processing through Particle Swarm Optimization for Target Motion Analysis with Bottom Bounce Underwater Acoustic Signals. Sensors.

[5] Kronhamn T. (1998). Bearings-only target motion analysis based on a multihypothesis Kalman filter and adaptive ownship motion control. IEE Proc.-Radar Sonar Navig..

[6] Mehrjouyan A., Alfi A. (2019). Robust adaptive unscented Kalman filter for bearings-only tracking in three dimensional case. Appl. Ocean. Res..

[7] Daowang F., Teng L., Tao H. (2010). Square-root second-order extended Kalman filter and its application in target motion analysis. IET Radar Sonar Navig..

[8] Liu J., Guo G. (2021). A Recursive Estimator for Pseudolinear Target Motion Analysis Using Multiple Hybrid Sensors. IEEE Trans. Instrum. Meas..

[9] Nardone S., Lindgren A., Gong K. (1984). Fundamental properties and performance of conventional bearings-only target motion analysis. IEEE Trans. Autom. Control.

[10] Blackman S., Popoli R. (1999). Design and Analysis of Modern Tracking Systems(Book).

[11] Wang Y., Bai Y., Wang X., Shan Y., Shui Y., Cui N., Li Y. (2021). Event-based distributed bias compensation pseudomeasurement information filter for 3D bearing-only target tracking. Aerosp. Sci. Technol..

[12] Voronina N.G., Shafranyuk A.V. (2021). Algorithm for constructing trajectories of maneuvering object based on bearing-only information using the Basis Pursuit method. J. Phys. Conf. Ser..

[13] Shalev H., Klein I. (2021). BOTNet: Deep Learning-Based Bearings-Only Tracking Using Multiple Passive Sensors. Sensors.

[14] Miller A.B., Miller B.M. (2018). Underwater target tracking using bearing-only measurements. J. Commun. Technol. Electron..

[15] Hou X., Zhou J., Yang Y., Yang L., Qiao G. (2021). Adaptive Two-Step Bearing-Only Underwater Uncooperative Target Tracking with Uncertain Underwater Disturbances. Entropy.

[16] Han X., Liu M., Zhang S., Zhang Q. (2019). A multi-node cooperative bearing-only target passive tracking algorithm via UWSNs. IEEE Sens. J..

[17] Wu K., Hu J., Lennox B., Arvin F. (2021). Finite-time bearing-only formation tracking of heterogeneous mobile robots with collision avoidance. IEEE Trans. Circuits Syst. II Express Briefs.

[18] Zhao S., Zhenhong L., Zhengtao D. (2019). Bearing-only formation tracking control of multiagent systems. IEEE Trans. Autom. Control.

[19] Hejazi F., Joneidi M., Rahnavard N. A tensor-based localization framework exploiting phase interferometry measurements. Proceedings of the 2020 IEEE International Radar Conference (RADAR).

[20] García-Fernández Á.F., Tronarp F., Särkkä S. (2019). Gaussian target tracking with direction-of-arrival von Mises–Fisher measurements. IEEE Trans. Signal Process..

[21] Doğançay K. (2015). 3D pseudolinear target motion analysis from angle measurements. IEEE Trans. Signal Process..

[22] Huang G., Zhou K., Trawny N., Roumeliotis S.I. (2015). A bank of maximum a posteriori (MAP) estimators for target tracking. IEEE Trans. Robot..

[23] Oh R., Shi Y., Choi J.W. (2021). A Hybrid Newton–Raphson and Particle Swarm Optimization Method for Target Motion Analysis by Batch Processing. Sensors.

[24] Santi F., Pastina D., Bucciarelli M. (2020). Experimental demonstration of ship target detection in GNSS-based passive radar combining target motion compensation and track-before-detect strategies. Sensors.

[25] Lebon A., Perez A.C., Jauffret C., Laneuville D. (2021). TMA from Cosines of Conical Angles Acquired by a Towed Array. Sensors.

[26] Bu S., Meng A., Zhou G. (2021). A New Pseudolinear Filter for Bearings-Only Tracking without Requirement of Bias Compensation. Sensors.

[27] Kirubarajan T., Bar-Shalom Y., Pattipati K.R., Kadar I. (2000). Ground target tracking with variable structure IMM estimator. IEEE Trans. Aerosp. Electron. Syst..

[28] Ulmke M., Koch W. (2006). Road-map assisted ground moving target tracking. IEEE Trans. Aerosp. Electron. Syst..

[29] Song D., Tharmarasa R., Florea M.C., Duclos-Hindie N., Fernando X.N., Kirubarajan T. (2019). Multi-vehicle tracking with microscopic traffic flow model-based particle filtering. Automatica.

[30] Oliva G., Panzieri S., Pascucci F., Setola R. (2015). Sensor networks localization: Extending trilateration via shadow edges. IEEE Trans. Autom. Control.

[31] Khatib O. (1986). Real-time obstacle avoidance for manipulators and mobile robots. Autonomous Robot Vehicles.

[32] Vadakkepat P., Tan K.C., Ming-Liang W. Evolutionary artificial potential fields and their application in real time robot path planning. Proceedings of the 2000 Congress on Evolutionary Computation. CEC00 (Cat. No. 00TH8512).

[33] Marzetta T.L. (1993). A simple derivation of the constrained multiple parameter Cramér-Rao bound. IEEE Trans. Signal Process..

[34] Benavoli A., Farina A., Ortenzi L. MLE in presence of equality and inequality nonlinear constraints for the ballistic target problem. Proceedings of the 2008 IEEE Radar Conference.

[35] Clarke F.H. (1990). Optimization and Nonsmooth Analysis.

[36] Mallick M. (2018). A note on bearing measurement model. Researchgate.

[37] Kay S.M. (1993). Fundamentals of Statistical Signal Processing.

[38] Doğançay K. (2005). On the efficiency of a bearings-only instrumental variable estimator for target motion analysis. Signal Process..

[39] Dorigo M., Birattari M., Stutzle T. (2006). Ant colony optimization. IEEE Comput. Intell. Mag..

[40] Schlueuter M., Gerdts M., Rückmann J.J. (2012). A numerical study of MIDACO on 100 MINLP benchmarks. Optimization.

[41] Schlueter M., Erb S.O., Gerdts M., Kemble S., Rückmann J.J. (2013). MIDACO on MINLP space applications. Adv. Space Res..

[42] Deb K., Pratap A., Agarwal S., Meyarivan T. (2002). A fast and elitist multiobjective genetic algorithm: NSGA-II. IEEE Trans. Evol. Comput..

[43] Oliva G., Rikos A.I., Hadjicostis C.N., Gasparri A. (2019). Distributed flow network balancing with minimal effort. IEEE Trans. Autom. Control.

[44] Aidala V.J. (1979). Kalman filter behavior in bearings-only tracking applications. IEEE Trans. Aerosp. Electron. Syst..

[45] Lingren A.G., Gong K.F. (1978). Position and velocity estimation via bearing observations. IEEE Trans. Aerosp. Electron. Syst..

[46] Farina A., Benvenuti D., Ristic B. (2002). A comparative study of the Benes filtering problem. Signal Process..

[47] Clark J., Vinter R., Yaqoob M. (2007). Shifted Rayleigh filter: A new algorithm for bearings-only tracking. IEEE Trans. Aerosp. Electron. Syst..

